# Assessing the Knowledge of Environmental Risk Factors for Cancer among the UAE Population: A Pilot Study

**DOI:** 10.3390/ijerph17092984

**Published:** 2020-04-25

**Authors:** Samrein B.M. Ahmed, Sara Amer, Amal Hussein, Drishti D. Kampani, Nour Al Hasham, Mohamed M. Assker, Nour Shawa, Dima Saleh, Khalid O. Alfarouk

**Affiliations:** 1 College of medicine, University of Sharjah, Sharjah 27272, UAE; MD_Saleema@outlook.com (S.A.); amalmh@sharjah.ac.ae (A.H.); U16103939@sharjah.ac.ae (D.D.K.); Nour_alhasham@outlook.com (N.A.H.); U17103019@sharjah.ac.ae (M.M.A.); U17100648@sharjah.ac.ae (D.S.); 2 Sharjah Institute for Medical Research, University of Sharjah, Sharjah 27272, UAE; nourshawa.1994@gmail.com; 3 Alfarouk Biomedical Research LLC, Temple Terrace, FL 33617, USA; khaliomer@gmail.com

**Keywords:** cancer, environment, risk factors, UAE population, knowledge

## Abstract

The incidence of cancer is increasing worldwide as well as in the United Arab Emirates (UAE). Currently, researchers are advocating not only for prevention programs but also for early detection. In this study, we aimed to assess the general awareness of cancer among the UAE population, with a focus on environmental risk factors. A descriptive cross-sectional design was employed, and a structured questionnaire was used to collect data from 385 participants. A total of 91.2% of the study population identified cancer as the leading cause of death, while 64.6% of the subjects were able to identify the key causes of cancer. A total of 87.3% and 70.5% of the participants were able to define tobacco and alcohol, respectively, as cancer-causing agents. Most of the study population failed to identify cancer-related infectious agents and incense smoke as carcinogens. Respondents in the medical professions had the highest knowledge score when compared with respondents with a non-medical profession and unemployed participants (*p* < 0.0005). To fill the gaps in cancer-related knowledge, participants were asked about their preferred method for cancer education, and 83.9% of the participants favored the media as a source of information. Conclusively, our findings indicated a gap in cancer knowledge among UAE residents, which highlights the importance of educational campaigns by health authorities; a follow-up study evaluating the success of educational campaigns is also warranted.

## 1. Background

Cancer is one of the leading causes of mortality worldwide. Global estimates show that the total incidence of cancer was 18.1 million with 9.6 million deaths in 2018, making it an important public health concern [1]. In the United Arab Emirates (UAE) alone, cancer is the third leading cause of death, preceded by cardiovascular disease and injury [2]. Despite advancements and improved clinical approaches to cancer treatment, resistance of cancer cells is inevitable. Therefore, scientists are proposing to combat cancer by either refining screening methods or developing protection strategies against cancer-causing agents [3].

Cancer etiology is attributed to (a) non-preventable genetic factors and (b) preventable environmental factors. These factors include occupational exposure, infectious diseases, air pollution, and ultraviolet (UV) radiation, among others. The substances that contribute to cancer development are called carcinogens, which act by damaging DNA and altering cellular machinery [4].

The significant increase in cancer burden worldwide has been linked to several factors, including an increase in cancer-causing behaviors and frequent exposure to cancer-causing agents. The World Health Organization (WHO) states that 30%–50% of cancers can be prevented by avoiding exposure to a carcinogen, adopting a healthier lifestyle and implementing evidence-based prevention strategies [5]. In the UAE, according to information published on the UAE governmental portal, approximately 4500 new cancer cases are reported each year [6].

Due to changes in economic and lifestyle trends, many disease patterns have shifted, including those of cancer. Primary prevention through environmental interventions has become the mainstay for reducing cancer burden. Cessation of tobacco and alcohol consumption remains the most common prevention recommendation worldwide [7]. Additionally, a case-control study by Tse and coinvestigators linked incense smoke to lung cancer [8]. Other important carcinogens that substantially influence cancer risk include bisphenol A, phthalates and naphthalene [9,10,11]. Moreover, commonly found substances such as household cleaning products, plastic containers, indoor combustion, soot and wood dust are reportedly carcinogenic [12].

Diet is also thought to play a crucial role in cancer development. Various food contaminants and food components have been linked to cancer pathogenesis. Examples of these carcinogens include aflatoxin [13,14] and processed meat [15]. Acrylamide is a dietary compound that is considered to have carcinogenic potential, and its mutational signature has been reported [16]. In addition, evidence from rats and mice indicated that saccharin has carcinogenic effects [17]. However, the carcinogenic potential of food-related agents depends on the individual’s susceptibility to developing cancer and their level of exposure.

It is estimated that occupational hazards can expose individuals to more than half of the chemicals classified as carcinogens. Based on the data provided by the Cancer Research UK, 4% of cancers are due to health hazards at work. Additionally, according to the International Agency for Research on Cancer, over 120 chemical or biological agents are classified as carcinogens [18]. Among these carcinogens are diesel exhaust, nickel, pesticides, asbestos, silica, wood dust and solar radiation [19]. Some common cancers associated with occupational carcinogen exposure are leukemia and lung cancer [20,21].

In addition to the abovementioned environmental carcinogens, biological carcinogens such as human papilloma virus, Epstein–Barr virus, hepatitis B and C and Helicobacter pylori have been associated with cancer incidence [22,23,24].

In the UAE, several programs, including the National Periodic Health Screening Program and Cancer Screening Initiative, were launched in 2015 by the Ministry of Health and Prevention to increase public awareness of cancer and the importance of its early detection through regular medical check-ups for more effective treatments [25]. However, little is known about the knowledge level or attitudes of the public towards cancer risk factors and exposure to carcinogens.

Because the UAE has stated measures regarding health safety for different occupations [26,27], in the present study, we aimed to assess the knowledge of the United Arab Emirates general population regarding environmental carcinogens. This will ultimately help in increasing the UAE community’s awareness about environmental carcinogens and thus help to establish goal-oriented campaigns that aim to reduce cancer burden locally by targeting and minimizing weaknesses in public knowledge. Additionally, we aimed to assess the knowledge and awareness of cancer among UAE populations with various educational levels, different age groups, and diverse occupational backgrounds.

## 2. Materials and Methods

To estimate the prevalence of knowledge regarding environmental risk factors among our study population, a descriptive cross-sectional design was used. The inclusion criteria included UAE residents aged 18 and above who were able to communicate in either English or Arabic, which are the most common languages in the UAE. Adults over the age of 18 who neither resided in the UAE at the time of the study, nor spoke English or Arabic were excluded from the study. The recruitment of the participants took place from February–May 2019.

A nonprobability convenience method was used to recruit subjects from public areas, such as parks, malls, and residential compounds. Because no similar study was conducted in the region, an expected proportion of study participants knowledgeable about the environmental risk factors for cancer was set at 50% to achieve the maximum sample size. A margin error of 5% was considered for the sample size calculation. The following equation was employed to calculate the sample size: $n=\frac{\left(4p\left(1-p\right)\right)}{SE^{2}}$, where *n* is the sample size, *p* is the expected prevalence and SE is the sampling error. Accordingly, 385 subjects were targeted.

A self-administered structured questionnaire was developed based on information acquired from identified sources and published cancer factors as well as global and local statistics, which are all cited in this manuscript. The questionnaire included a total of 41 questions. The questionnaire included items related to demographics (6), general knowledge of cancer (5), knowledge related to environmental carcinogens (27), sources of cancer-related knowledge (2) and knowledge related to cancer prevention (1). The questionnaire was self-administered, and the data collectors were available to clarify any ambiguity. The questionnaire was also pilot tested to ensure face and content validity.

Prior to data collection, the data collectors ensured that the method of data collection was consistent and standardized. The data collectors verbally explained the project and its outcomes to the participants. Moreover, it was clearly stated in the information sheet as well as verbally that completing the questionnaire indicates the agreement to join the study. No signature was taken to ensure the anonymity of the study. Additionally, the data collector explained that the questionnaire would take only 10 min to complete; the time was allotted after the pilot testing. The collected data were available only to the investigators to ensure confidentiality. This study was approved by the Research Ethics Committee at the University of Sharjah (Ref. No. REC-18-12-02-01) before the study commenced.

After data entry and cleaning, 32 questionnaires were excluded because of incomplete and missing data. The data were analyzed electronically using IBM SPSS, version 25 (IBM Corp., Armonk, NY, USA). The study subgroups included age, level of education, occupation field, country of origin, UAE residence duration, and residence in different emirates. Categorical data are summarized as frequencies and percentages, and scale data are summarized as the mean and standard deviation when normally distributed and the median and interquartile range when skewed. The normality of the scale data was tested visually using Q_Q plots and statistically using the Kolmogorov–Smirnov test. Parametric statistical tests such as Analysis of Variance (ANOVA) test and Student’s *t*-test were performed to compare mean knowledge scores between groups when parametric assumptions were met. Otherwise, the Kruskal-Wallis and Mann-Whitney U test non-parametric tests were performed when knowledge score data showed a skewed distribution in at least one of the compared groups. Stepwise multiple linear regression (MLR) was performed to identify significant predictors of the cancer-related knowledge score. Variables entered in the model were defined as those associated with the dependent variable in the bivariate analysis, with a *p*-value less than or equal to 0.20. Assumptions of MLR were all tested prior to performing the analysis. Collinearity was tested using tolerance and variance inflation factor, and multivariate outliers were checked using Mahalanobis distance. Equality of variance was tested using Levene’s test. Dummy variables were created for categorical variables with more than two groups. A *p*-value less than 0.05 was considered statistically significant.

## 3. Results

### 3.1. Demographic Data of the Study Population

In this study, 353 participants were included in our final analysis. Age was categorized into 4 groups: 18–24 years of age (39.4%), 25–35 years of age (23.2%), 36–45 years of age (17.6%), and above 45 years of age (19.8%). Of all study participants, 84.4% had bachelor’s degrees or above, while 15.6% completed elementary, intermediate, or high school education. Of the participants, 72.5% were employed, 12.5% were employees in the medical field, and 64% were employed in a non-medical field. Most of the study population had been UAE residents for more than 5 years (82.4%, *n* = 291). Participants resided in the seven emirates of the UAE, where the highest portion (43.1%) of the study sample resided in Sharjah.

### 3.2. Assessing the Study Population’s General Knowledge about Cancer

Of the participants, 98.3% (*n* = 347) had heard of cancer, of whom 92.8% (*n* = 322) believed that cancer could lead to death, and 81.6% (*n* = 283) considered cancer to be a preventable disease.

**Figure 1:**
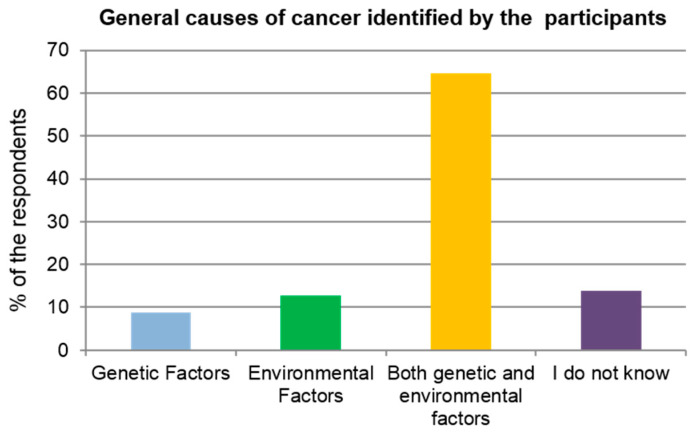
General causes of cancer identified by the study population.

**Figure 2:**
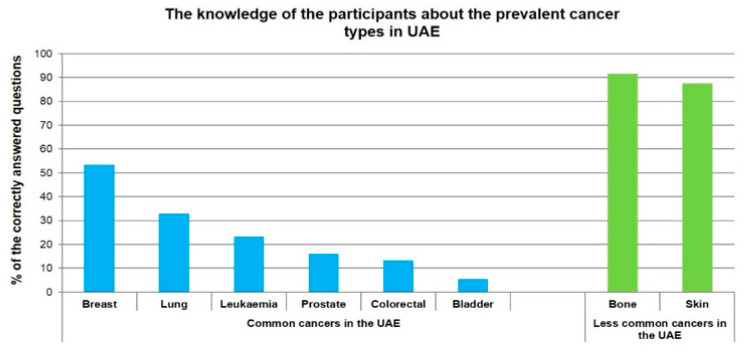
The knowledge of the participants regarding the prevalent cancer types in the UAE.

Figure 1 and Figure 2 illustrate the general knowledge and awareness of cancer among the study population. Two thirds of the study subjects were correctly aware that cancers can be caused by both genetic and environmental factors (Figure 1). 

According to the WHO 2018 statistics, breast, colorectal, prostate, leukemia, bladder and lung cancers are among the most prevalent cancers in the UAE [28]. Upon questioning the participants about the predominant types of cancer in the UAE, 26.6% (*n* = 94) of the respondents stated that they did not know about cancer prevalence in the country. Out of the six common types of cancer in UAE, breast cancer was correctly identified as common by more than half the respondents. Leukemia, prostate, and colorectal cancer were least recognized as prevalent cancers with only 23.2%, 15.9% and 13.3% identifying them, respectively. Despite the poor knowledge and awareness of prevalent cancers, the study population correctly identified the non-prevalent cancers, such as bone and skin cancers, as uncommon. Overall, the study population demonstrated a lack of knowledge regarding the prevalent cancers in the UAE.

### 3.3. Knowledge of Cancer Risk Factors among the Study Population

**Figure 3:**
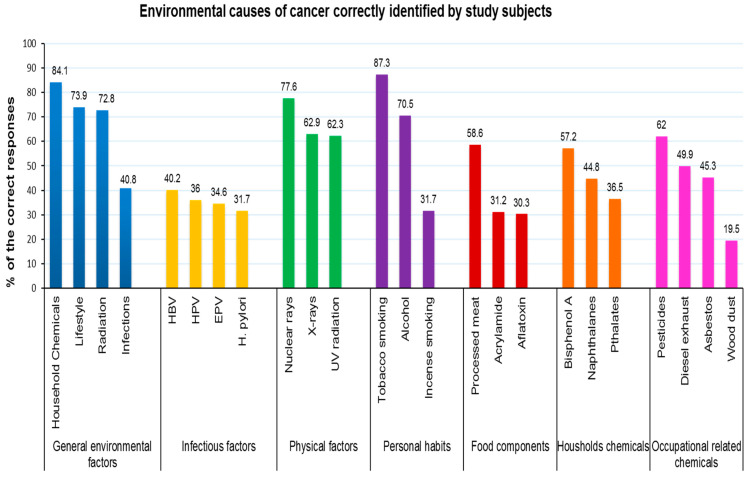
Environmental causes of cancer correctly identified by the study subjects. HBV: Hepatitis B virus; HPV: Human Papilloma virus; EPV: Epstein–Barr virus; H. pylori: Helicobacter pylori.

Figure 3 demonstrates the level of knowledge about the leading risk factors for cancer. The impact of chemicals, lifestyle, and radiation factors on cancer development was correctly identified but poor knowledge was observed regarding tumor-associated microorganisms. Less than half of the subjects (40.8%, *n* = 144) believed that infections are a possible leading cause of cancer.

Additionally, low awareness was found among the respondents regarding the effects of some food-related components and contaminants, such as saccharin (36.3%), aflatoxin (30.3%) and acrylamide (31.2%). 

The best identified cancer causes by our population were physical factors and lifestyle causes. A total of 77.6% of the participants identified nuclear radiation as a risk for cancer development; tobacco smoking and alcohol consumption were positively identified as causes by 87.3% and 70.5% of the study population, respectively. Conclusively, many of the study participants were able to identify smoking, alcohol, and nuclear radiation as cancer-causing factors, but disappointingly, a large section of the respondents failed to categorize infections, some food-related contaminants/components, incense smoke, and occupation-associated carcinogens as risk factors.

### 3.4. The Knowledge Scores among the Subgroups According to Different Study Variables

The cancer-related knowledge score of all study participants ranged from 0 to 100%, with a mean of 51% (SD = 20.5) and a median of 52% (IQR = 25.9). The score had a non-normal distribution as indicated by the Kolmogorov–Smirnov test (KS = 0.054, df = 353, *p*-value = 0.016). Knowledge scores were compared between the different groups of the study variables (age, level of education, occupation, or residency). The two variables that were significantly associated with the cancer-related knowledge score were occupation and duration of residency in the UAE. Respondents in the medical professions had the highest knowledge score (64.2%) compared with those of participants with non-medical professions (50.6%) and unemployed respondents (45.3%) (*p* < 0.0005).

Those who were UAE residents for less than 5 years showed lower cancer-related knowledge than those who resided in the UAE for more than 5 years (44.4% and 51.9%, respectively, *p* = 0.012). None of the other variables, including age, education, country of origin, and Emirate, showed a significant relationship with the cancer-related knowledge score (Table 1).

The stepwise MLR model included the variables age, level of education, occupation, and residency duration in the UAE. The model was significant in predicting 7.8% of the total variability in the cancer-related knowledge score (F = 14.717, *p* < 0.0005). Two predictors were identified by the regression model: occupation and age. Having a medical occupation predicted an increase in the knowledge score of 15.2% compared to the scores of those who were unemployed (*p* < 0.0005), while being above 45 years old was associated with a 7.0% increase in the knowledge score compared to the scores of those who were 18 to 24 years old (*p* = 0.008), when keeping all other variables constant. Accordingly, it was inferred that participants with a medical profession positively impacted the knowledge score of the study population, while surprisingly, participants with more than 5 years of residence in the UAE exhibited better knowledge than those who had been in the UAE for fewer than 5 years.

### 3.5. Sources of Cancer Knowledge

Most of the participants (81.9%) considered the sources of media, such as TV, radio, and internet, to be their source of knowledge, while a small percentage of the subjects (20.7%) claimed that they obtained health-related information from health care centers. To determine the best method to increase cancer knowledge among the UAE population, the participants were asked what source they would prefer to use to increase their knowledge of cancer. Among the media, campaigns and hospitals, 83.9% of participants selected the media as their preferred source of knowledge, followed by campaigns and hospitals.

### 3.6. Desirable Methods to Prevent Poor Cancer Outcomes Identified by the Study Participants

We were also interested in assessing the awareness of the study population regarding different cancer prevention approaches. Regular medical check-ups and healthy lifestyle and diet were the most popular methods among our respondents (67.4% and 59.8%, respectively). However, just under 27% of the respondents thought that vaccines could help with cancer prevention.

## 4. Discussion

Various environmental factors that are related to lifestyle, personal habits, food and occupation have been found to be linked with cancer development [5]. Exposure to certain environmental factors related to cancer development is considered a modifiable cancer risk factor [5]. Moreover, since cancer treatment is challenging for advanced-stage cancer, early diagnosis as well as the enhancement of preventive measures against cancer are a prerequisite to improve disease outcomes [3]. In this pilot study, we aimed to assess the general knowledge about cancer as well as to assess the awareness of the UAE population regarding some of the identified environmental risk factors using a cross-sectional study design. More than half of the study participants were able to relate cancer development to both environmental and genetic factors. Although the study population was aware of some of the environmental risk factors, they exhibited a lack of knowledge about others, which indicates that further comprehensive studies are required to develop and promote existing awareness programs.

### 4.1. Knowledge of Cancer as a Deadly Disease Is High

In this study, cancer was identified as a leading cause of death by 91.2% of the participants. This finding is consistent with previously published studies reporting that general populations strongly believe that cancer is a fatal disease, particularly in the Middle Eastern region, where a diagnosis of cancer is highly stigmatized [29,30].

Furthermore, a study conducted in the US further confirmed this observation, in which people held strongly fatalistic beliefs about cancer. These beliefs have caused people to disengage from cancer prevention strategies because they believe that nothing can be done to prevent it [30]. These attitudes may have led to an increase in cancer incidence. Additionally, people are less likely to participate in screening tests for fear of a positive result [31].

Another study conducted in Ireland concluded that 15% of the study population agreed with the statement that if cancer was present in their family, “there was nothing a person could do to reduce their own personal cancer risk” [32].

On a positive note, although unexpected, 80.2% of the respondents concurred that cancer was a preventable disease, indicating that the public was familiar with awareness campaigns.

### 4.2. Knowledge of Common Cancers among the Study Population

In this study, we found that 53.5% of the participants correctly responded that breast cancer is a common type of cancer. This result is comparable to findings from a previous study in which UAE female residents reported having limited knowledge of breast cancer screening tests [33]. Despite the Pink Caravan campaign initiative [34], public awareness requires further improvement.

Regrettably, our study population showed a lack of knowledge when asked about other types of cancer. For example, only 13.3% (*n* = 47) of the respondents agreed that colorectal cancer is a common cancer in the UAE, despite its predominance in males in the UAE [28]. In September 2018, the Ministry of Health-UAE launched a colorectal cancer (CRC) awareness program for early detection and prevention [35]. However, our study sample recruitment was performed in the first half of 2019, which might explain the lack of awareness about CRC. This might indicate the importance of a future follow-up study to evaluate the success of the awareness campaign.

An important question about cancer awareness is how to identify the people with the least amount of knowledge. In this study, the respondents whose professions were related to the medical field showed better knowledge scores than the rest of the respondents (*p* < 0.0005), implying a need to diversify health awareness campaigns to appeal to people from different backgrounds. This finding is in agreement with an Indian study that reported that women in the medical field have better breast cancer awareness than women employed in non-medical professions when both groups had received the same level of education [36].

Additionally, it is worth noting that the level of education had no correlation with the cancer knowledge score. This result implies that participants with lower educational backgrounds have comparable knowledge to those with higher education backgrounds. A study by Gurdal et al. inferred that women are required to increase their awareness about breast cancer regardless of their level of education [37]. One recommendation would be to consider the introduction of cancer-oriented awareness campaigns at all levels for better coverage. Accordingly, health care facilities as well as the government need to work together to promote awareness of other common types of cancer that are prevalent in the country.

### 4.3. Knowledge of Environmental Risk Factors

In this study, more than half of the study population (64.6%) agreed that cancer is caused by both genetic and environmental factors. Interestingly, previous studies have indicated that people strongly emphasize only family history and genetic factors as causes of cancer [38,39]. In addition, our findings demonstrate that participants were able to correctly report some environmental factors.

A study conducted in England reported that 91% of the study population was able to correctly report cancer risk factors [38]. Similarly, in an American study, the population sample identified cancer risk factors, yet a lack of knowledge regarding the association between the major risk factor and the corresponding cancer was noted in the study [39]. These two studies showed better cancer-related knowledge in some of the Western countries than that of our study population. This indeed indicates that more effort is required to promote cancer-related knowledge among UAE residents, even though more than half of the study population was able to relate both genetic and environmental factors to cancer.

Infections were the least recognized cancer risk factor in the study population. This observation is consistent with a study conducted in Latin America, in which 47% of the participants were able to identify Human papillomavirus (HPV) as a risk factor for cancer [40]. In contrast, a study in Japan demonstrated that the public had better knowledge of cancer-associated infections as a risk factor for cancer than of other risk factors [41]. Thus, it is worth increasing awareness about infectious agents as a cause of cancer, particularly HPV, because it was recently reported that cervical cancer-related deaths are increasing in the UAE [42].

In our study population, smoking (87.3%) and alcohol consumption (70.5%) were primarily identified as environmental risk factors for cancer. In contrast, a previous report that was conducted in Germany showed that only 47% of the study population was able to link alcohol to cancer [43]. The better knowledge in our study population could be because of the sociocultural stigma associated with alcohol consumption in the region. This indeed discourages the practice and promotes the idea that alcohol is unhealthy [44]. The expected relatively high knowledge of the link between smoking and cancer is due to the issued federal law No. 15 of 2009 regarding tobacco control, which was agreed upon by the government and departments of health and smoking cessation clinics that are easily accessible across the country [45]. Moreover, the anti-smoking campaign agenda was announced in 2010 by hospitals and the UAE Ministry of Health targeting the whole population of the UAE [46,47]. It is worth noting that the health hazards of smoking are a global concern, and all sources of media demonstrate this explicitly, which might explain the noted level of smoking-related knowledge among the participants. Consistent with our study, a previous Polish study on tobacco health risks revealed that 92% of the study participants recognized the link between tobacco smoking and cancer [48].

Expectedly, incense smoke (bakhour) was least identified as a possible cancer-causing agent in our study population (31.7%). This result highlights the fact that although people are aware of common risk factors, it is important to educate the general public about some traditional practices that might lead to cancer. An interesting study by Yvonne et al. showed that the burning of indoor incense was associated with oral microbiota composition among 303 Emirati adults [49], which implies the effect of incense smoke on the health of the Emirati population. Although this study highlighted the negative effect of incense smoke on the health of the Emirati population, no studies have demonstrated the link of leukemia and lung cancer, two of the most prevalent cancers in the UAE, with household incense. Performing such a study will help in providing evidence of the link between the use of the incense and cancer development among the UAE population. Hence, preventive health measures can be issued by the health authority to enhance awareness about incense smoke as a possible risk of cancer among the UAE population.

Furthermore, the study population demonstrated a lack of knowledge regarding carcinogenic food contaminants/additives such as saccharine, pesticides, bisphenol A, aflatoxin, and acrylamide. Assessing population awareness of exposure to pesticides among other chemical carcinogens in the Hail region-KSA revealed an extreme lack of awareness that made an awareness campaign a necessity in the Hail region [50].

### 4.4. Sources of Knowledge and Methods to Prevent Poor Cancer Outcomes

We found that our study population favored being educated about health and prevention via the media. A study on the use of social media to raise awareness about breast cancer suggested that social media is a preferable method for raising breast cancer awareness [51]. Having mentioned this, the employment of the media should be tailored to the age group, level of education, language, and cultural barriers. Moreover, because almost half of the study participants were between 18 and 35 years of age, it was not surprising that the media was the dominant choice for gaining knowledge.

Counterintuitively, our results showed that a residency of more than 5 years in the UAE was associated with a higher knowledge score (*p* = 0.012). This result may be explained in part by the fact that the major health authorities in the UAE started intensive cancer awareness campaigns in 2013 [52]. The effectiveness and evaluation of the launched awareness programs and campaigns require further detailed studies that is beyond the scope of this study.

Regular medical check-ups and living a healthy lifestyle were considered methods to reduce poor cancer outcomes by more than half of the study population. On the other hand, most of the study population (74%) did not consider vaccination to be a preventive measure against cancer. Nevertheless, this finding is consistent with the demonstrated lack of knowledge regarding cancer-related infections. This further emphasizes the need to develop campaigns to raise awareness among the population regarding the microorganisms associated with cancer development, particularly cervical cancer, as its incidence is increasing in the UAE [42].

### 4.5. Study Limitations

This research used a convenience sampling method, which may affect the generalizability of the results. In the conservative society of the UAE, home visits are not appropriate, and physical mail is not a popular mode of communication. Thus, the sex demographics of the respondents were not ascertained in this study. In addition, the age demographic of the study may not be perfectly representative of the UAE population. According to 2018 UAE population estimates, 42.17% of the total adult population is between 25 and 34 years of age; however, in this study, only 23.2% of the participants were in this age range. Thus, our findings may underestimate the magnitude of age-related and education-related differences in the knowledge and attitudes related to common cancers and environmental risk factors related to cancer development.

## 5. Conclusions

A lack of awareness about common yet risky exposure to substances may be an important and overlooked impediment to national cancer prevention strategies. The findings solidly support the development of intensive cancer-relevant information campaigns in educational institutions, workplaces, and society. These campaigns should particularly raise cancer awareness regarding cancer-associated infections as well as sources of indoor pollution, such as incense smoke, which is associated with increased cancer risk and cervical cancer, leukemia, and lung cancer, respectively. Further studies could assess the specific effects of community- and government-led cancer prevention initiatives on awareness levels. Future studies could also investigate whether sex differences exist in terms of the knowledge of common cancers and awareness of available screening programs. Moreover, the prevalence of carcinogenic exposure within society, in terms of duration and levels, could be investigated further.

Moreover, these campaigns and awareness programs should promote knowledge about the common cancers in the country and their available screening programs as well as their associated risk factors.

## Figures and Tables

**Table 1:** Cancer-related Knowledge Score by Demographic characteristics of study participants.

	*n*	Score (%)	Test Value	*p*-Value
Age				
18–24	139	51.9	6.681 *	0.083
25–35	82	48.1		
36–45	62	48.1		
>45	70	55.6		
Level of education				
Below University	55	48.1	7015.0 *	0.089
University and Above	298	51.9		
Occupational field				
Medical	44	64.2	13.264 **	*<0.0005*
Non-Medical	226	50.6		
Unemployed	83	45.3		
Country of origin				
Middle East	275	51.9	10635 *	0.910
Others	78	48.1		
UAE Residency duration				
Less than 5 years	62	44.4	7187.0 *	*0.012*
5 years and above	291	51.9		
Emirate				
Abu Dhabi	53	48.1	1.127 *	0.770
Dubai	78	51.9		
Sharjah	152	48.1		
Northern Emirates	70	51.9		

* Test is Kruskal-Wallis or Mann-Whitney U test. ** Test is ANOVA or independent *t*-test.

## Data Availability

All data generated or analyzed during this study are included in this published article.

## Participant Information Sheet (PIS)

The PIS was presented upon approaching the participants. Agreement to fill the questionnaire indicated the agreement of the participants to join the study; this was clearly mentioned in the PIS.

## Abbreviations

UV	Ultraviolet radiation
DNA	Deoxyribonucleic acid
UAE	United Arab Emirates
UK	United Kingdom
US	United States
